# Testing Chemical Safety: What Is Needed to Ensure the Widespread Application of Non-animal Approaches?

**DOI:** 10.1371/journal.pbio.1002156

**Published:** 2015-05-27

**Authors:** Natalie Burden, Fiona Sewell, Kathryn Chapman

**Affiliations:** NC3Rs, London, United Kingdom

## Abstract

Scientists face growing pressure to move away from using traditional animal toxicity tests to determine whether manufactured chemicals are safe. Numerous ethical, scientific, business, and legislative incentives will help to drive this shift. However, a number of hurdles must be overcome in the coming years before non-animal methods are adopted into widespread practice, particularly from regulatory, scientific, and global perspectives. Several initiatives are nevertheless underway that promise to increase the confidence in newer alternative methods, which will support the move towards a future in which less data from animal tests is required in the assessment of chemical safety.

## Abstract 2

Scientists face growing pressures to move away from using traditional animal toxicity tests to determine whether manufactured chemicals are safe. This essay explores the incentives, hurdles, and ongoing initiatives towards a reduced reliance on such tests.

## Introduction

It is over 50 years since Russell and Burch first introduced the concept of the 3Rs—the reduction, refinement, and replacement of the use of animals in research and testing [1]. Since then, there have been increasing requirements for scientists using animals in their daily work to consider and incorporate these guiding principles into their research. A recent surge in initiatives from various organisations around the world is helping to create an environment in which the consideration and application of the 3Rs is becoming more widespread. There are the beginnings of a move towards a future in which more human-relevant, non-animal systems are used in the study of biological processes and in the early stages of the development of novel pharmaceutical compounds.

One area that remains heavily reliant on animal models, however, is chemical safety assessment, in which toxicity tests are carried out to ascertain whether manufactured products pose a threat to the health of humans or the environment. This continued reliance is largely due to the considerable debate about whether non-animal methods will ever truly be capable of predicting the effects of chemicals on a whole, intact organism. There is also a perception that only safety assessments based on animal data can convincingly address the strong societal concerns around human exposure to environmental chemicals, such as those that may affect the reproductive system. However, the increasing pressure to reduce the use of animals for scientific purposes, in alignment with the principles of the 3Rs, and the recent bans on the testing of cosmetics on animals in Europe [2] and other geographical regions, means that the use of non-animal approaches is no longer just a desire, but a necessity. The types of such approaches already in development and use for the prediction of toxic effects include cell-based (in vitro) methods (such as stem cell technologies, tissue engineering, and organs-on-chips), next-generation sequencing and ‘omics technologies (including genomics, epigenomics, and proteomics), and computational (in silico) modelling techniques.

Here, we detail incentives to make the transition away from traditionally used toxicity tests, challenges that must be met before animal tests can be confidently replaced with newer alternative approaches, and highlight some of the initiatives being undertaken to help the toxicology field embrace the 3Rs.

## What Are the Incentives to Move Away from Animal Toxicity Tests?

One obvious motivation for reducing, refining, and replacing the use of animals in chemical toxicity testing is ethical; toxicity tests can be associated with high levels of suffering (for example, in acute toxicity tests where death is the main endpoint), and/or large numbers of animals are used. For example, it was estimated when the European chemicals regulation REACH (Registration, Evaluation, Authorisation, and restriction of CHemicals [3]) came into force in 2009, that millions of animals would be used in the testing of 30,000 chemicals [4]. However, there are several additional, and often overlooked, drivers and benefits associated with the use of non-animal approaches in this area.

### Scientific Drivers

The development and application of alternative methods, and the questioning of traditional approaches, has potential to drive better scientific practice. One of the problems with using laboratory animals (usually rodents) to test for the harmful effects of chemicals, is that the scientific question that must be answered is not how safe is a chemical for a rat or a mouse, but is it acceptable for a human to be exposed to it? There are obvious inherent differences in the underlying biology of humans and other mammals, and therefore humans are not always well represented by traditional test species (e.g., see [5]). It is also uncertain whether variability within human populations, due to either genetic or environmental factors, can be captured sufficiently within laboratory animal models. Therefore, it is important to consider how well the different predictions of toxicity made using animals translate to the human situation, and in some instances, it may be necessary to re-assess the test organism that is used. For example, shifting towards the use of human cells to study chemically induced perturbations could improve the predictability and human relevance of toxicity tests, the value of which has already been recognised for the assessment of drug efficacy [6,7]. The multitude of existing data on human-specific chemical effects (e.g., available from post-marketing monitoring studies, poison centres, and databases such as the United States Environmental Protection Agency [EPA]'s Integrated Risk Information System [IRIS]) could also now be utilised to build more predictive mathematical and computational modelling techniques, providing a more reliable means of flagging potential problem chemicals before they are ever exposed to biological tissue. On the other hand, there is also increasing evidence that non-mammalian species may be useful in predicting toxic effects in humans, provided there is sufficient conservation of the pathways in question; for a more detailed review see [8]. Non-animal test methods will also come in useful when addressing specific questions about the mechanisms by which chemicals exert their biological effects, and some of these methods have contributed towards our understanding of complex relationships between dose and biological response. One example of this is when greater effects occur at low concentrations compared with higher ones, a phenomenon that has been observed for a subset of chemicals [9].

### Business Drivers

There are also business drivers for chemical companies to reduce their animal use. Animal experiments are resource intensive, particularly considering the housing and staffing costs involved, and therefore, development costs could potentially be reduced if fewer animal tests are carried out. The opportunities are not only in replacing animal tests with non-animal technologies; reducing and refining the required animal tests can also have significant business benefits. For example, blood sampling of animals using sophisticated microsampling techniques (where microlitres of blood are taken and can be accurately analysed) illustrates how the costs can be reduced through the use of fewer animals, less staffing resource, and smaller amounts of candidate chemicals compared with the typical methods [10,11]. This is an approach that has value across multiple sectors of the chemicals industry—in the testing of both industrial and agro-chemicals, as well as pharmaceuticals.

### Regulatory Drivers

There are also legal reasons why companies must reconsider their use of animals in toxicity testing. Within Europe, the cosmetics industry can no longer manufacture or market products that have undergone animal tests [2], and this has been followed by a geographical ban in India [12], with Australia and the US possibly following suit (www.alp.org.au/cosmeticstesting; www.congress.gov/bill/113th-congress/house-bill/4148). Other regulations that govern the production and sale of chemicals, particularly in Europe, also stipulate that animal studies should only be carried out as a last resort [3,13,14]. Considering that products are now developed for global marketing, businesses are being increasingly compelled to seek viable scientific alternatives to animal testing in order to meet these regional obligations.

### Opportunities to Improve and Refine Animal Studies

Finally, it is worth noting that while the computational and cell-based methods are being improved to ensure they are fit-for-purpose, there are various ways in which to generate better quality and/or more meaningful data within the currently used animal tests. In the previous microsampling example, any toxic effects observed in the animals can be directly related to the circulating concentrations of drug/chemical [10,11]. This contrasts to traditional toxicokinetics studies, in which large blood samples are taken from completely distinct groups of animals and toxic effects are not assessed. There are also opportunities to refine currently used animal tests; for example, through recent cross-company sharing of data on chemical toxicity, an objective positive predictive scoring system has been developed so that early signs of toxicity, rather than death, can be used as the readout in acute toxicity studies, in order to reduce the degree of suffering test animals experience [15]. It is also important that the reporting of research using animals continues to be improved, in order to maximise the utility of published information (e.g., to allow for systematic reviews and “evidence-based” approaches, as proposed in [16]), and to minimise the number of studies carried out unnecessarily.

## What Are the Barriers That Need to Be Overcome?

Although many sectors wish to transition to non-animal approaches, a desire that is becoming more realistic as a result of recent scientific advances, several barriers remain that must be overcome before they can be put into widespread practice.

### Limited Regulatory Acceptance of Alternative Approaches

Some of the barriers are related to the regulatory nature of toxicity tests. Decision makers working on behalf of regulatory bodies determine whether the safety of a chemical has been adequately assessed by companies through appropriate scientific tests, in line with the legislative requirements. These regulators must therefore be certain that the alternative methods are as reliable and give the same level of information as the traditionally accepted tests. There can sometimes be reluctance among regulators to embrace new technologies and approaches, and little flexibility in adapting to alternative methods, with more traditional methods being preferred and perceived as less “risky.”

Another factor precluding regulatory acceptance is the uncertainty around how to handle the data generated from alternative methods and how it should be interpreted—for example, data generated using ‘omics technologies can be very complex and require a high level of expertise to interpret them in the context of human risk assessment. This is in contrast with the extensive historical experience in the interpretation of data generated using traditional, generally animal, methods. There can also be ambiguity in the interpretation of the regulatory guidelines that describe the information requirements, occasionally leading to the perception (from both the regulatory bodies and scientists) that a specific amount of animal data is required, when it is not. One example of this is a perceived need to include recovery animals in all pharmaceutical toxicity studies [17]. Sometimes data is generated to “tick boxes” even though there is no scientific rationale for its inclusion. Redundancy in some animal tests has been proven, such as the one-year dog study for agro-chemicals that has been shown through retrospective data analysis not to add any value to safety assessment over and above the information provided by a similar 90-day study—as such, this test has now been removed from many regional requirements [18,19]. It has also been shown in some instances that additional toxicity tests that examine the effects of the same chemical through different exposure routes provide little added value for hazard assessment purposes compared with assessing effects via only one relevant route of administration [20], mitigating the need to carry out multiple studies for the same chemical.

There are ongoing global efforts, co-ordinated by organisations such as the International Cooperation on Alternative Test Methods (ICATM), the Interagency Coordinating Committee on the Validation of Alternative Methods (ICCVAM), the European Union Reference Laboratory for alternatives to animal testing (EURL-ECVAM), and the Japanese Center for the Validation of Alternative Methods (JaCVAM), which aim to ensure, through formalised processes, that newly developed methods are robust, reproducible, and fit-for-purpose, from a scientific and regulatory perspective. Whilst these processes will certainly help to contribute towards greater regulatory acceptance, such validation exercises can be costly and time consuming. Therefore, a more streamlined approach is needed to ensure the reliability and relevance of alternative methods in development and use, whether for regulatory or non-regulatory purposes, such as the vast number of high-throughput in vitro screens being employed in the US ToxCast programme [21]. The establishment of regulatory agency “safe harbours,” such as that provided by the Food and Drug Administration (FDA), is another potential means by which regulators could assess non-standard data in parallel with that generated by traditional methods, to build awareness and facilitate greater acceptance in the long term.

### Lack of Globally Harmonised Requirements

The removal of the one-year dog study for agro-chemicals from some but not all regional requirements also highlights the need for improved global harmonisation of the data requirements. It can be the case that, to enable global marketing, animal studies are carried out to meet the requirements of a single country or region, despite other countries not requiring that data. From a business perspective, companies often take the most risk-averse approach so as not to endanger the regulatory acceptance of their safety assessments. Improved communication between regulators, industry researchers, and honest brokers will be invaluable in guaranteeing the necessary shift in attitude towards the acceptance of science-driven processes, and a real cultural change at the regulatory level.

### Scientific Barriers

There are also many scientific aspects that must be addressed before the community as a whole is comfortable with using non-animal approaches in standard practice. There remains much uncertainty around proving that they are at least as accurate and sensitive in their predictions as the tests they set out to replace. There is also recognition that alternative methods cannot realistically replace the traditional tests on a like-for-like basis, and therefore a consensus must be agreed on how to integrate all the potential new methods available. There also remain gaps in our scientific knowledge that will delay the development and application of alternative approaches, particularly in areas of toxicology that are extremely complex, such as the assessment of mixtures effects and in developmental toxicity testing. Therefore, future investment is needed to further our understanding of the science that underlies these effects before there can begin to be sufficient confidence in the utility of alternative approaches for these types of assessment. A key need for the future is the building and maintaining of cross-sector collaborations and the routine sharing of data to successfully implement change.

## Current Status: The Reduction and Replacement of Animal Use in Practice

There is evidence that companies are starting to use non-animal methods more widely [22], although it is not clear at this point if they are being used in place of or in addition to animal tests. It is important to recognise that using these tools alongside each other is a vital first step towards the commonplace use of alternatives, and will allow for comparisons to be made between the traditional and newer approaches. This comparison, in turn, will help to reassure regulators and companies alike of the utility and applicability of the alternative approaches.

### Opportunities to Waive Animal Tests

Within many regulatory frameworks, the option now exists to waive particular tests if human exposure to a chemical is unlikely (e.g., [23]) and/or if it is scientifically justified. For example, existing data can be taken into consideration and used to inform decisions about whether further animal testing is required. Waiving of tests may also be possible in certain situations in which redundancy has been proven, as described above (e.g., if a chemical’s acute toxicity has been determined through testing following its oral administration, it may be possible to avoid additional testing through a second [dermal] route [20]). The option to potentially waive tests emphasises the need for scientists to carefully plan and consider their study designs in the most logical and informed manner, on a case-by-case basis for each chemical. This will help to ensure that when animal tests are still required, they are not carried out unnecessarily.

### Investment into the Development and Application of Non-animal Alternatives

The recognition that there are many potential benefits to moving away from the traditional tests used in chemical safety assessment is evident in the fact that several large-scale, international research initiatives are currently underway, attempting to overcome some of the barriers mentioned above. For example, in the US, the Tox21 programme (www.epa.gov/ncct/Tox21), a multi-agency effort involving the US National Institutes of Health (NIH)—National Institute of Environmental Health Sciences and NIH—National Center for Advancing Translational Sciences, EPA, and FDA, has been established to use high-throughput robotics technology in the screening of thousands of chemicals for potential toxicity, to use this screening data to predict the potential toxicity of chemicals, and to develop a cost-effective approach for prioritising thousands of chemicals for toxicity testing. Within Europe, the SEURAT-1 cluster (www.seurat-1.eu), which was set up with funding from the European Commission and the cosmetics industry, is formed of six projects that aim to fill current gaps in scientific knowledge and accelerate the development of non-animal test methods, with a focus on the complex area of repeated dose toxicity. A truly global initiative, which was launched in 2012, is the Organisation for Economic Cooperation and Development (OECD)’s Adverse Outcome Pathways programme (www.oecd.org/chemicalsafety/testing/adverse-outcome-pathways-molecular-screening-and-toxicogenomics.htm), which aims to use crowd-sourcing through a web-based platform to bring together all knowledge on how chemicals can induce adverse effects.

Along with these collaborative efforts, there has also been targeted investment into the development of non-animal technologies, for example, Innovate UK’s 2014 and 2015 funding calls around advancing the development and application of non-animal technologies, the Wyss Institute’s Enabling Technology Platform to develop technologies applicable to biomimetic microsystems (including organ-on-chip, wyss.harvard.edu/viewpage/enabling-technology-platforms/enabling-technology-platforms), and the European Commission’s €88 million (approximately US$100 million) Horizon 2020 funding call around “New approaches to improve predictive human safety testing” (http://ec.europa.eu/research/participants/portal/desktop/en/opportunities/h2020/topics/9078-phc-33-2015.html). These initiatives will visibly shape the landscape towards more useable non-animal technologies and approaches; however, the next step will require taking these advances and applying them in practice, which involves ensuring that the developments are disseminated to the wider scientific community and regulatory bodies, by companies and scientific organisations such as the NC3Rs, to ensure that novel approaches are taken up and accepted. It will be critical to engage regulators in the early stages of research and development of novel alternative approaches, to capture their input and ensure that the correct questions are being asked of the work; i.e., how will the regulatory concerns be addressed, and how will the data be handled and interpreted in a regulatory context? The ultimate aim over the coming years will be for the regulatory agencies to adopt and integrate the alternative methodologies, once demonstrated as fit-for-purpose, into their policy and guidance, which will allow the risk assessors to consider their use more widely. The data requirements will likely need to be devised on a case-by case basis to ensure that best scientific practice drives the risk assessments, particularly considering that several tests may be needed to provide an answer that one animal test would have provided in the past; this has been recognised at the international level, with the OECD now embarking on activities that support Integrated Approaches to Testing and Assessment (IATA).

## Concluding Remarks

Recent years have seen a turning point in our ability to consider the risk of chemicals to humans by using more data from non-animal technologies. There is great potential to apply these scientific and technological advances to reduce our reliance on animal tests and also to establish toxicity screens that bear more human and real-life relevance. While the community works together towards greater regulatory acceptance of non-traditional approaches, short-term gains in the reduction of the numbers of animals and the suffering experienced by test animals can be achieved through the application of novel techniques and better study design. There is also scope for non-animal techniques to be utilised in the early screening of newly developed chemicals, to prioritise candidate selection, inform chemical development (for example, changing the chemistry of the molecule prior to animal studies), and help avoid the testing of harmful chemicals in animals during later stages of development. The knowledge and experience gained from these experiments will be invaluable in the shift towards regulatory acceptance. The numerous joint research initiatives underway hold real promise to provide the scientific foundation that is required to increase confidence in alternative methods. The industry and regulatory communities must now work together to ensure that these achievements are applied in practice, so that chemical safety assessment strategies can be improved and animal use can be reduced.
